# Comparison of partitioned survival modeling with state transition modeling approaches with or without consideration of brain metastasis: a case study of Osimertinib versus pemetrexed-platinum

**DOI:** 10.1186/s12885-024-11971-x

**Published:** 2024-02-09

**Authors:** Yoon-Bo Shim, Byeong-Chan Oh, Eui-Kyung Lee, Mi-Hai Park

**Affiliations:** School of Pharmacy, Sungkyunkwan University, 2066 Seobu-ro, Jangan-gu, Suwon, Gyeonggi- do Republic of Korea

**Keywords:** Partitioned survival model, Markov model, Cost-effectiveness analysis, Quality-adjusted life-year

## Abstract

**Background:**

The partitioned survival model (PSM) and the state transition model (STM) are widely used in cost-effectiveness analyses of anticancer drugs. Using different modeling approaches with or without consideration of brain metastasis, we compared the quality-adjusted life-year (QALY) estimates of Osimertinib and pemetrexed-platinum in advanced non-small cell lung cancer with epidermal growth factor receptor mutations.

**Methods:**

We constructed three economic models using parametric curves fitted to patient-level data from the National Health Insurance Review and Assessment claims database from 2009 to 2020. PSM and 3-health state transition model (3-STM) consist of three health states: progression-free, post-progression, and death. The 5-health state transition model (5-STM) has two additional health states (brain metastasis with continuing initial therapy, and with subsequent therapy). Time-dependent transition probabilities were calculated in the state transition models. The incremental life-year (LY) and QALY between the Osimertinib and pemetrexed-platinum cohorts for each modeling approach were estimated over seven years.

**Results:**

The PSM and 3-STM produced similar incremental LY (0.889 and 0.899, respectively) and QALY (0.827 and 0.840, respectively). However, 5-STM, which considered brain metastasis as separate health states, yielded a slightly higher incremental LY (0.910) but lower incremental QALY (0.695) than PSM and 3-STM.

**Conclusions:**

Our findings indicate that incorporating additional health states such as brain metastases into economic models can have a considerable impact on incremental QALY estimates. To ensure appropriate health technology assessment decisions, comparison and justification of different modeling approaches are recommended in the economic evaluation of anticancer drugs.

**Supplementary Information:**

The online version contains supplementary material available at 10.1186/s12885-024-11971-x.

## Background

In cost-effectiveness analyses of anticancer drugs, partitioned survival models (PSM) and state transition models (STM) are the most common model structures applied for oncology drugs [1]. PSM has the advantage of simplicity as it directly uses each survival curve (e.g., progression free survival (PFS) and overall survival (OS)) to estimate the proportion of state membership. However, PSM has limited ability to capture the complexity of a disease with multiple stages because it can only be applied when patients move forward through a set of health states without backward transitions. The STM can be a primary alternative to the PSM. In the STM, the number of patients in each state is dictated by the transition probabilities between mutually exclusive health states. This approach is advantageous owing to its flexibility, although challenges exist in terms of finding robust transition probabilities, especially with multiple health states. It requires more complex methods to reflect time-dependencies in event rates. The STM allows for more health states to be constructed than in PSM, enabling the model to apparently reflect the natural history of the disease. Therefore, it is recommended to use STM alongside PSM to explore uncertainties in the extrapolation period according to the National Institute for Clinical and Care Excellence (NICE) Technical Support Document 19 [2].

The 3-health state transition model (3-STM) is conventionally established with health states of “progression-free (PF),” “post-progression (PP),” and “death.” However, the course of disease sometimes includes heterogeneous health states that lead to higher or lower costs and quality of life (QoL). In these cases, the need for additional health states is highlighted to capture the disease course. However, there is a lack of research on how much the additional health states affect the results of the cost-effective analysis in oncology. To deal with these issues, we developed a 5-health state transition model (5-STM) considering additional health states with heterogeneity.

We conducted a case-study study on Osimertinib, the preferred regimen for first and subsequent line of therapy in epidermal growth factor receptor (EGFR)-mutated non-small cell lung cancer (NSCLC) with or without brain metastases [3]. In South Korea, patients with T790M mutation are eligible for reimbursement of Osimertinib if they experienced disease progression following the prior administration of an EGFR-tyrosine kinase inhibitor (TKI). Patients with EGFR-mutated NSCLC frequently develop brain metastasis [4, 5], which can lead to a higher economic burden, poorer prognosis, and lower QoL compared to metastasis in other sites [6–8]. In addition, patients with EGFR-mutated NSCLC can continue to use Osimertinib even if brain metastasis progresses [3]. The continuation of Osimertinib treatment despite brain metastasis was observed in the phase 3 AURA3 trial, where the median time to first subsequent therapy or death (16.0 months) was longer than median PFS (10.1 months) [9, 10]. Given these differences, we considered brain metastasis a heterogeneous event from other progressions and included two additional health states: brain metastasis with continuing initial therapy and with subsequent therapy.

This study evaluated the life-years (LY) and quality-adjusted life-years (QALY) estimates for Osimertinib and pemetrexed plus platinum chemotherapy (PPC) as an applied example to explore structural uncertainty. We developed three economic models (PSM, 3-STM, and 5-STM) for patients with EGFR-mutated advanced NSCLC, whose disease had progressed on first-line EGFR-TKI. To estimate the impact of different modeling approaches, we compared PSM and 3-STM. We also built a 5-STM to investigate how adding heterogeneous health states to the 3-STM affects the results of LY and QALY.

## Methods

### Data source and extraction of patient-level data

Patient-level data were extracted by analyzing national claims data from the Health Insurance Review and Assessment Service (HIRA) database in South Korea. We used data from January 1, 2009, to October 30, 2020 (Study period). The analysis of national claims data was approved by the Institutional Review Board of Sungkyunkwan University (IRB No. SKKU 2021-01-026) and details of the database are described in Supplementary Methods S1.

We identified patients with EGFR-mutated advanced NSCLC whose disease had progressed on first-line EGFR-TKI. Patients who switched therapy to osimertinib were included in the Osimertinib cohort, whereas those who switched therapy to PPC were included in the PPC cohort. Both cohorts were followed from the date of the treatment switch (index date) until either death or the end of the study period (30 October 2020). The study design is illustrated in S1 Fig. To alleviate the imbalance between the two cohorts, propensity score matching was conducted with the greedy 1:1 matching algorithm. The standardized mean difference was estimated to check whether both cohorts were balanced. Details regarding the patient selection and propensity score-matching are presented in Supplementary Methods S2.

OS, time to next treatment (TTNT), and time to brain metastasis were estimated in the Osimertinib and PPC cohorts. The OS was calculated from the index date to the date of death, and patients who were alive were censored at the dataset cut-off date. The method of extracting the date of death is described in Supplementary Methods S3. TTNT was used as a proxy for PFS, which was calculated from the index date to the date of the next treatment or death because the HIRA database does not provide information about disease progression. Patients who did not receive subsequent treatment or who were alive at the end of the study period were censored. Time to brain metastasis was defined as that from the index date to the diagnosis. The diagnosis of brain metastasis was defined as having at least one inpatient or two outpatient claims with International Classification of Disease-10th revision (ICD-10) code C793 (secondary malignant neoplasm of the brain and cerebral meninges).

All analyses of the national claims data were conducted using the SAS Enterprise Guide version 6.1 (SAS Institute, Inc., Cary, North Carolina, USA) and R version 3.5.1 (The R Foundation for Statistical Computing, Vienna, Austria). The SAS Enterprise Guide software was used for data management and analyses, and R was used to create Kaplan–Meier survival curves.

### Economic models

We developed three economic models (PSM, 3-STM, and 5-STM) to compare the LY and QALY estimates in EGFR-mutated advanced NSCLC patients whose disease had progressed on first-line EGFR-TKI. The time horizon of the models was set to seven years given that the proportion of patients alive at such time was less than 0.1 in the extrapolated OS curves. Sensitivity analyses were conducted using the 5- and 10-year time horizons. A 3-week cycle length was selected considering that PPC is administered every three weeks. LY and QALY were discounted using a rate of 4.5%, according to the health technology assessment (HTA) requirements of South Korea. All models were constructed using Microsoft Excel® (Microsoft Corp., Redmond, Washington, USA).

#### Partitioned survival model (PSM)

**Fig. 1:**
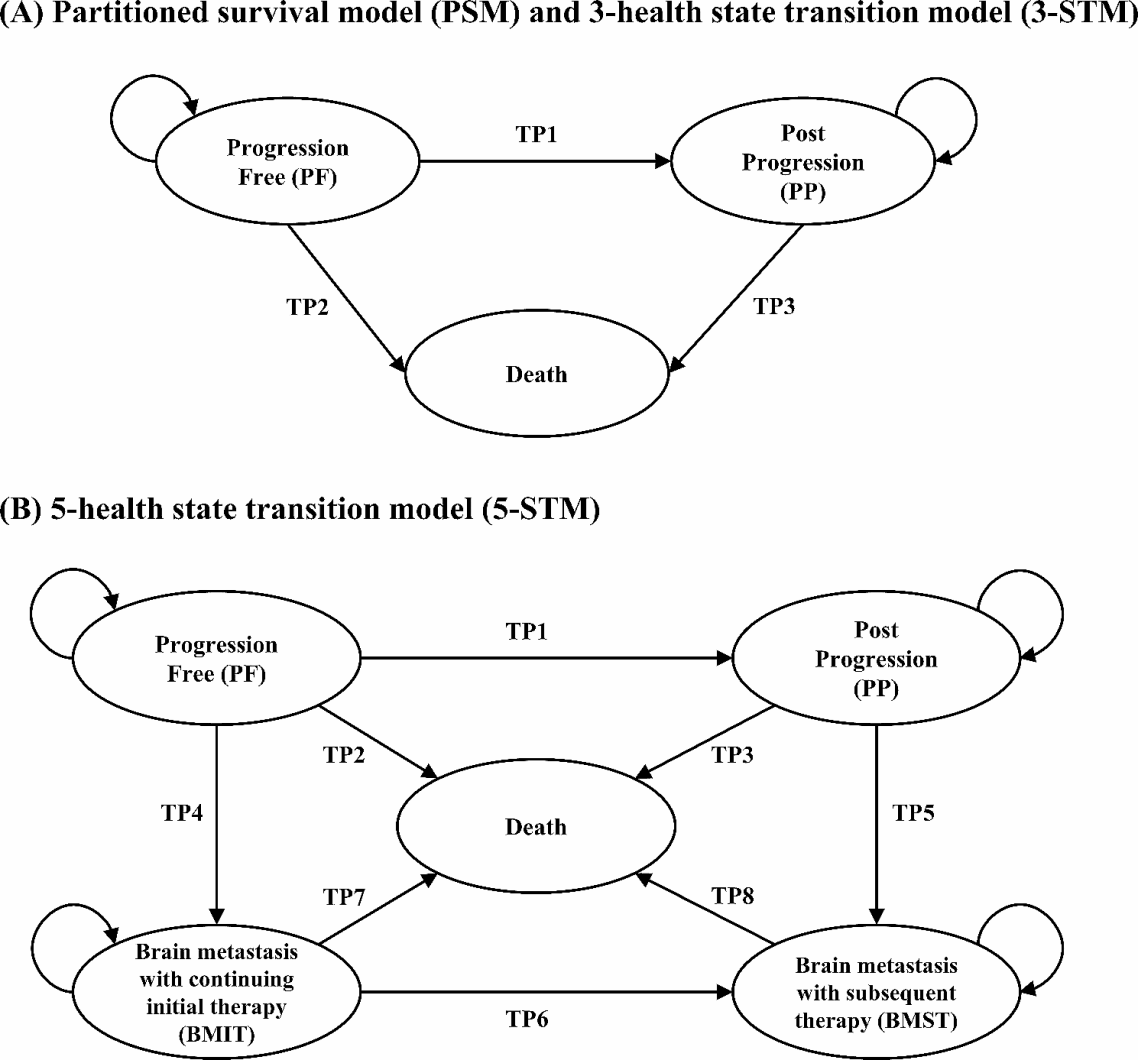
Model structure

As illustrated in Fig. 1, the PSM consists of three health states: Progression free, Post-progression, and Death. The proportion of patients in each health state over time was directly determined using the areas under the parametric OS and TTNT curves. The proportion of patients who had died at each time point was calculated as one minus the OS curve. The difference between the OS and TTNT curves was considered as the proportion of patients who experienced progression. The TTNT curve (a proxy for the PFS curve) directly provided the proportion of patients remaining in the PF health state.

#### 3-health state transition model (3-STM)

The 3-STM is characterized by three health states: Progression free (starting state), Post-progression, and Death (Fig. 1). The 3-STM was constructed using time-dependent transition probabilities between the three health states. A total of three transition probabilities were estimated: from PF to PP (TP_1_), from PF to death (TP_2_), and from PP to death (TP_3_). The transition probabilities for each health state were derived from parametric survival curves fitted to the patient-level data from the survival analysis. The survival functions and equations for TP_1_, TP_2_, and TP_3_ to calculate the transition probabilities are described in the Supplementary Methods S4, and S1 Table. Tunnel states were used to track the time spent in a PP health state [11].

#### 5-health state transition model (5-STM)

In 5-STM, two additional health states were constructed: brain metastasis with continuing initial therapy (BMIT) and brain metastasis with subsequent therapy (BMST), as illustrated in Fig. 1. The inclusion of these health states was based on clinical practice guidelines that recommend that Osimertinib can be continued even if brain metastasis occurs [3]. Patients with a history of brain metastasis as of the index date started the model in the BMIT health state, whereas the other patients started the model in the PF health state. A total of eight time-dependent transition probabilities were calculated between the five health states: from PF to PP (TP_1_), from PF to death (TP_2_), from PP to death (TP_3_), from PF to BMIT (TP_4_), from PP to BMST (TP_5_), from BMIT to BMST (TP_6_), from BMIT to death (TP_7_), and from BMST to death (TP_8_). If NSCLC patients maintained the initial therapy (Osimertinib or PPC) despite brain metastasis, the transition from PF to BMIT health state occurred (TP_4_). Post-progression patients whose disease had been regarded as progressed by the initiation of subsequent therapy were transited to the BMST health state when they experienced brain metastasis (TP_5_). The patients in the BMIT health state transitioned to the BMST health state when subsequent therapy was initiated (TP_6_). The transition probabilities for each health state were estimated using patient-level data derived from survival analysis. The survival functions and equations for TP_1_–TP_8_ used to calculate the transition probabilities are described in Supplementary Methods S4, and S2 Table. The tunnel states were used to track the time spent in the PP, BMIT, and BMST health states.

### Modeling survival

To extrapolate patient survival, parametric survival models were fitted to the following time-to-event data extracted by analyzing national claims data as described in Sect. 2.1: OS, TTNT, time to death from PF, time to death from PP, TTNT from PF, time to brain metastasis from PF, time to brain metastasis from PP, TTNT in metastasis with continuing initial therapy, time to death in metastasis with continuing initial therapy, and time to death in metastasis with subsequent therapy. The process for fitting the parametric survival models was based on guidance from the Decision Support Unit of NICE [12]. The proportional hazards assumption was tested using log-cumulative hazard plots, Schoenfeld residual plots, and the global test. Dependent models were fitted when the assumption of proportional hazards held, and independent models were fitted when such assumption did not hold. A total of six parametric distributions were considered: Exponential, Weibull, Gompertz, Lognormal, Loglogistic, and Generalized gamma distributions. The best-fitting model was chosen by considering Akaike’s information criterion (AIC), AIC with correction (AICc), Bayesian information criterion (BIC), and visual inspection. The selected parametric survival curves were used to populate the PSM, 3-STM, and 5-STM. All the endpoints were analyzed independently, and parametric survival modeling was conducted using the *flexsurv* package in the software R version 3.5.1 (The R Foundation for Statistical Computing, Vienna, Austria).

### Utility inputs

The utility weights are sourced from two previous studies. Jiang et al. [13] reported utilities of patients with EGFR-mutated advanced NSCLC collected using the Euroqol 5-dimension (EQ-5D) instrument. We applied utilities of Osimertinib and chemotherapy groups reported in the literature. For the utility of brain metastasis health states, we used the values reported by Roughley et al. [8] Additionally, as reported by Jiang et al. [13], we applied a 0.042 decrease in utility as the disease progressed. The utility weights used in economic models are presented in Table 1. Alternative utilities of PF and PP health states were examined in the sensitivity analyses (S3 Table).

**Table 1:** Model input parameters and parametric distributions

Model input	Osimertinib	PPC	Source
**Patient characteristics**			
% of females	61.5		HIRA data
Age at the start, years	64.9		HIRA data
**Utilities**			
Progression-free	0.800	0.730	Jiang et al.
Post-progression	0.758	0.688	Jiang et al.
Brain metastasis with continuing initial therapy	0.520	0.520	Roughley et al.
Brain metastasis with subsequent therapy	0.478	0.478	Roughley et al. Jiang et al.
**Parametric distributions for PSM**			
Time to next treatment	Log-logistic	Log-normal	Best fit
Overall survival	Generalized gamma	Generalized gamma	Best fit
**Parametric distributions for 3-STM**			
Time to next treatment	Log-logistic	Log-normal	Best fit
Overall survival	Generalized gamma	Generalized gamma	Best fit
Time to death from PF	Log-normal	Log-normal	Best fit
Time to death from PP	Log-normal	Log-logistic	Best fit
**Parametric distributions for 5-STM**			
Time to next treatment from PF	Generalized gamma	Generalized gamma	Best fit
Time to death from PF	Log-normal	Log-normal	Best fit
Time to death from PP	Log-normal	Log-logistic	Best fit
Time to brain metastasis from PF	Generalized gamma	Log-normal	Best fit
Time to brain metastasis from PP	Exponential	Log-normal	Best fit
Time to next treatment in metastasis with continuing initial therapy	Exponential	Weibull	Best fit
Time to death in metastasis with continuing initial therapy	Log-normal	Log-normal	Best fit
Time to death in metastasis with subsequent therapy	Log-normal	Exponential	Best fit

PF, progression-free; PP, post-progression; PPC, pemetrexed plus platinum chemotherapy; 3-STM, 3-health state transition model; 5-STM, 5-health state transition model

### Outcomes of each modeling approach

We calculated incremental LY and QALY between Osimertinib and PPC cohorts for each modeling approach. We also estimated LY and QALY divided by each health state. To understand the differences in the modeling approaches, the distribution of patients in each health state for Osimertinib and PPC over time was illustrated. For PSM and 3-STM, the probability of residing in the PF, PP, and death health states were plotted over time. For 5-STM, the probabilities of residing in PF and BMIT health states were combined, as well as those of residing in PP and BMST health states. The area under the curve (AUC) was calculated with the trapezoidal rule.

## Results

**Fig. 2:**
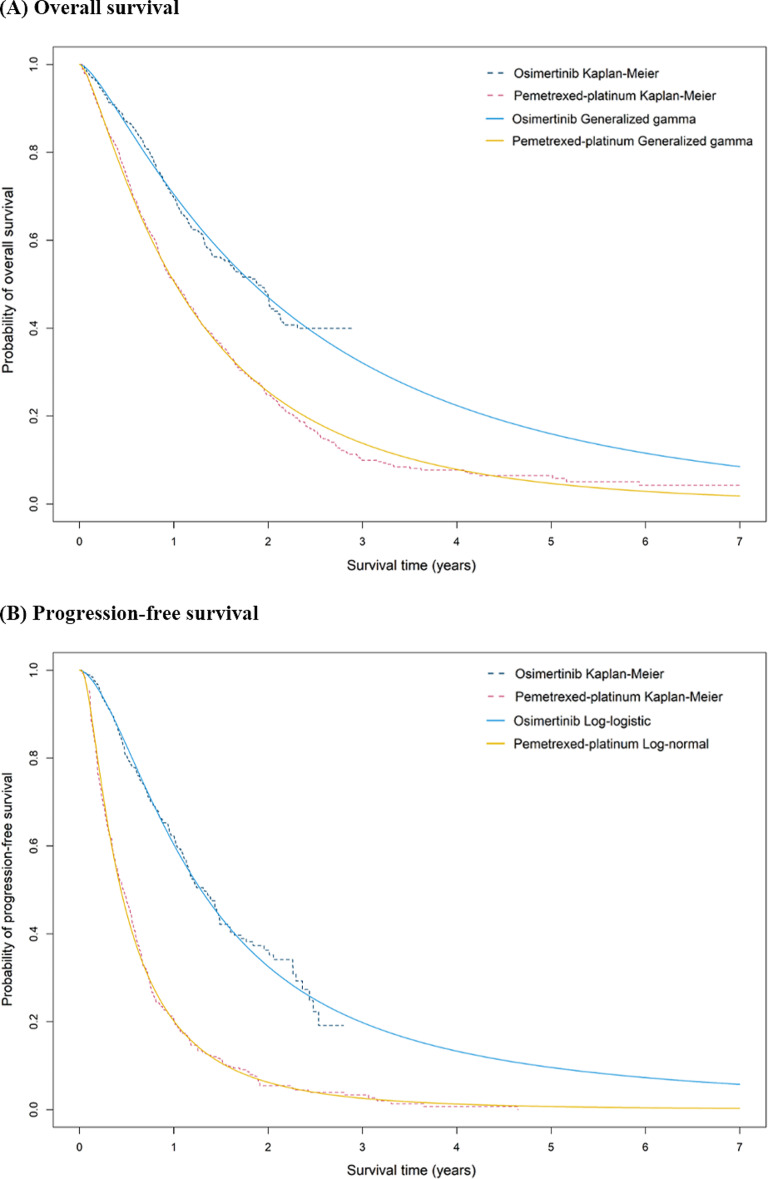
Comparison of Kaplan-Meier data and parametric extrapolation model. (A) overall survival; (B) Time to next treatment

In the HIRA database, 3,491 patients with EGFR-mutated advanced NSCLC used Osimertinib or PPC as second-line palliative therapy. After 1:1 matching on the propensity score, 735 patients were included in each of the Osimertinib and PPC cohorts (S2 Fig.). The baseline characteristics of the propensity score-matched cohort were well-balanced, with standardized mean differences of less than 0.1 (S4 Table). The Kaplan–Meier data and fitted parametric curves for OS and TTNT analyzed in the Osimertinib and PPC cohorts are presented in Fig. 2. Model input parameters, including the best-fitting distributions for each survival curve, are listed in Table 1.

**Fig. 3:**
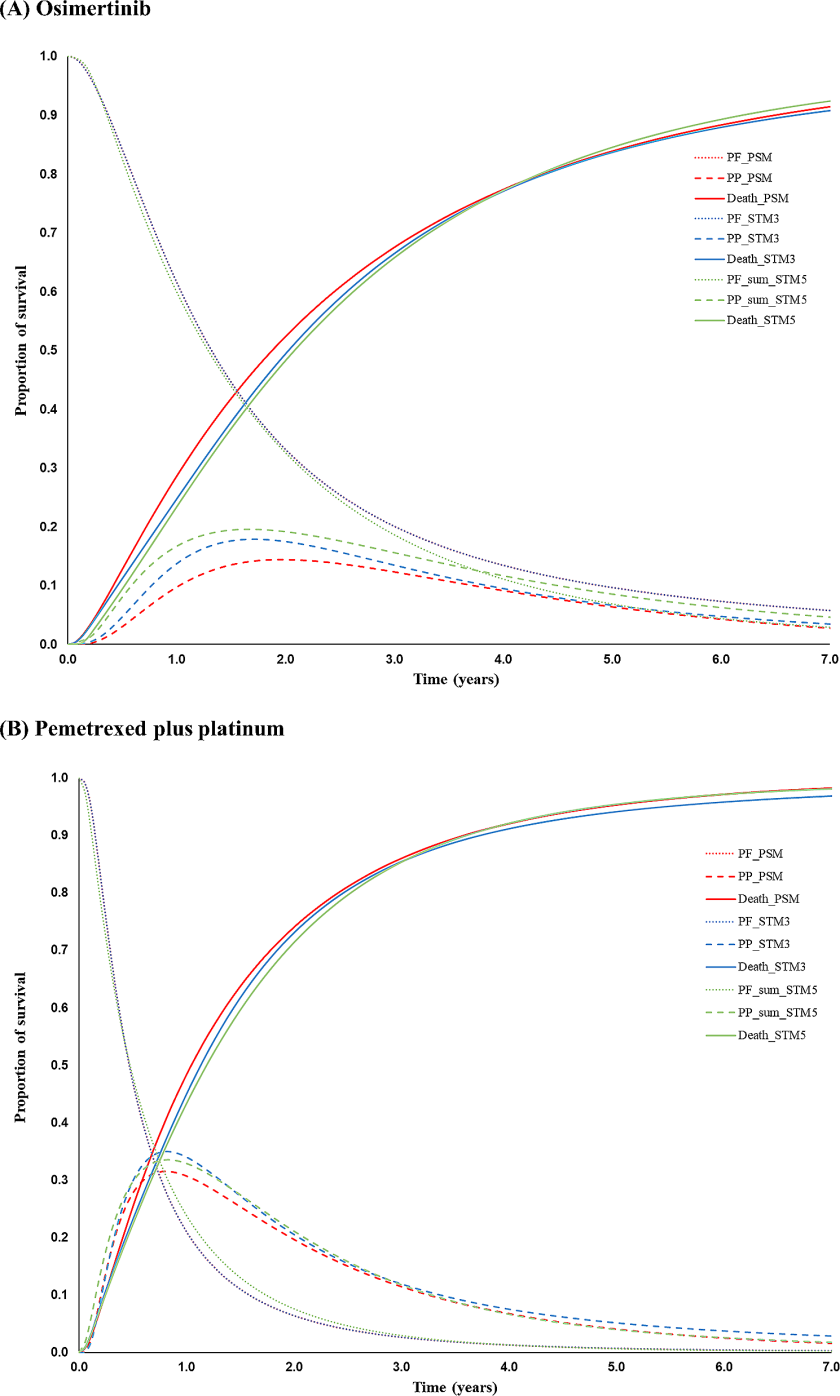
Distribution of patients in each health state for osimertinib (**A**) and pemetrexed plus platinum (**B**) over time. PF, progression-free; PP, post-progression; PSM, partitioned survival model; STM3, 3-health state transition model; STM5, 5-health state transition mode. * Progression-free curves of PSM and STM3 overlap

The distribution of the patients in each health state over time for each model structure is illustrated in Fig. 3, and the corresponding AUC values are presented in S5 Table. Estimates of the difference in mean LY between Osimertinib and PPC over seven years are reported in Table 2, and were 0.89, 0.90, and 0.91 in PSM, 3-STM, and 5-STM, respectively. The mean QALYs for were 1.90 (PSM), 1.96 (3-STM), and 1.73 (5-STM) for Osimertinib, and 1.07 (PSM), 1.12 (3-STM), and 1.03 (5-STM) for PPC. The incremental QALYs for each model are presented in Table 2, which were 0.83, 0.84, and 0.70 for PSM, 3-STM, and 5-STM, respectively. The incremental QALY for 5-STM was smaller than that for PSM and 3-STM, with differences of -16% and − 17%, respectively. The estimated LYs and QALYs divided by each health state in each model structure are reported in S6 Table. Although similar, PSM predicted slightly fewer LYs for both Osimertinib and PPC. The mean LYs for were 2.40 (PSM), 2.49 (3-STM), and 2.50 (5-STM) for Osimertinib, and 1.51 (PSM), 1.59 (3-STM), and 1.59 (5-STM) for PPC.

**Table 2:** Incremental LY and QALY estimates by each model type

	PSM	3-STM	5-STM
**Incremental LY**	**0.889**	**0.899**	**0.910**
Progression-free	1.136	1.136	0.689
Brain metastasis with continuing initial therapy	-	-	0.312
Post-progression	-0.247	-0.237	-0.193
Brain metastasis with subsequent therapy	-	-	0.101
**Incremental QALYs**	**0.827**	**0.840**	**0.695**
Progression-free	0.962	0.962	0.594
Brain metastasis with continuing initial therapy	-	-	0.162
Post-progression	-0.134	-0.121	-0.109
Brain metastasis with subsequent therapy	-	-	0.048

PSM, partitioned survival model; 3-STM, 3-health state transition model; 5-STM, 5-health state transition model; LY, life-year; QALY, quality-adjusted life-year

*Details may not add up to total due to rounding

The results of one-way sensitivity analyses are presented in Table 3 and S7 Table. The time horizon had the greatest impact on incremental QALYs, ranging from 0.71 to 0.91 (PSM), 0.73 to 0.92 (3-STM), and 0.61 to 0.74 (5-STM). The results were robust to changes in utility sources, exhibiting a less than 10% reduction in incremental QALYs.

**Table 3:** Results of one-way sensitivity analysis

	Incremental LY			Incremental QALY		
	PSM	3-STM	5-STM	PSM	3-STM	5-STM
**Base-case**	0.889	0.899	0.910	0.827	0.840	0.695
**Time horizon**						
5 years	0.745	0.769	0.779	0.710	0.732	0.614
10 years	0.991	0.994	0.991	0.910	0.920	0.744
**Discounting rate**						
3%	0.921	0.931	0.942	0.855	0.867	0.717
7.5%	0.831	0.842	0.852	0.778	0.792	0.656
**Utility**						
Bertranou et al.	0.889	0.899	0.910	0.758	0.765	0.644
AURA2 EQ-5D-5 L Crosswalk Values	0.889	0.899	0.910	0.753	0.760	0.639
AURA2 EQ-5D-5 L England Valuation Set Values	0.889	0.899	0.910	0.802	0.810	0.665

PSM, partitioned survival model; 3-STM, 3-health state transition model; 5-STM, 5-health state transition model; LY, life-year; QALY, quality-adjusted life-year

## Discussion

In this study, three economic models were constructed to compare the estimates of LY and QALY between the modeling approaches. Despite the different assumptions of the two modeling methods, PSM and 3-STM derived similar results for LY and QALY. In contrast, 5-STM, constructed to reflect the characteristics of Osimertinib, revealed similar incremental LY and lower incremental QALY than those in PSM and 3-STM.

The use of alternative modeling approaches between PSM and 3-STM using time-dependent transition probabilities had little impact on the incremental LY (0.889 versus 0.899) and QALY (0.827 versus 0.840). The difference in the results was attributed to the differences in the method of estimating the proportion of patients in each health state. PSM directly determined the proportion of patients in the death state using the areas under the OS curves. In contrast, 3-STM uses a set of time-dependent transition probabilities between three health states.

Several studies have reported the impact of different model structures within economic models of oncology drugs. Cranmer et al. compared PSM and STM and found that survival estimates were similar within the trial period, but differed after the end of follow-up [14]. Goeree et al. compared the modeling approaches of PSM and STM in patients with advanced NSCLC and reported similar results for LY and QALY, which is consistent with our results [15]. Smare et al. evaluated the survival outcomes of nivolumab and everolimus in renal cell carcinoma and reported differences in survival across three model structures [16]. Smare et al. reported that two variations of STM had higher incremental survival benefits compared with PSM of up to 14%. However, owing to the dissimilarity in the diseases and the longer time horizon a direct comparison with our study would be inappropriate.

In our study, there were considerable differences in incremental QALY (0.840 vs. 0.695) between the 3-STM and 5-STM groups. Incremental LY was greater in 5-STM, but incremental QALY in 3-STM was greater than that in 5-STM. This is likely due, in part, to the fact that patients who developed brain metastases but continued to use Osimertinib were categorized as being in a PFS health state at 3-STM, whereas at 5-STM, they were in a BMIT health state with lower utility. In addition, the impact of lower utility values for brain metastasis states would have been more reflected in the Osimertinib cohort, which survives longer in the brain metastasis states than in the PPC cohort. These results reflect the clinical reality that Osimertinib can be continued in EGFR-mutant NSCLC with brain metastases owing to improved blood-brain barrier penetration [17–19]. As 5-STM was larger in incremental LY, the results may have differed depending on the difference in utility between the presence and absence of brain metastasis.

Considering the impact on incremental QALY, our findings highlight the importance of selecting an appropriate model structure. It may be necessary to consider additional health states when heterogeneous conditions exist, and the choice of additional health states may vary depending on the type of cancer or treatment. Further research is needed to determine which model structure should be used and when, and a cost analysis should be included to determine the impact on the incremental cost-effectiveness ratio. When incorporating additional health states, cohort STM is suitable as long as the characteristics of the decision problem can be captured with a manageable number of health states [20]. While there is no absolute limit on the number of states in a cohort STM [21], if a large number of additional health states are required, it may be more appropriate to consider another approach, such as the individual-level STM. Also, the model structure and specifications of health states should reflect the understanding of the disease course and adequately capture the benefits of interventions [20].

The strength of this study is that we explored structural uncertainty with the direct calculation of transition probabilities by measuring the time to brain metastasis, which has not been reported in the clinical trial literature. We developed a 5-STM that reflects the disease course of EGFR-mutated advanced NSCLC and compared it with conventional PSM and 3-STM. Real-world clinical outcomes used in the economic models were estimated using a nationwide health insurance claims database covering nearly the entire Korean population. This can provide a more representative sample of patients, with enhanced generalizability, capturing a broader range of age groups and healthcare practices.

This study had several limitations. First, TTNT was used as a proxy for PFS because the HIRA database does not provide information on disease progression. There is a possibility of overestimating PFS given that the Osimertinib cohort exhibited a longer TTNT than PFS in the AURA3 trial [9, 10]. According to previous studies, the TTNT was similar to or exceeded real-world PFS and could be considered a proxy for real-world PFS, although further validation is needed [22–25]. It is noteworthy that patients not initiating subsequent treatment might introduce bias, especially in cases where individuals with compromised health conditions refrain from treatment, contributing to potential overestimation of PFS. Second, the date of death was extracted using the operational definition described in the Methods section. However, we adapted an operational definition validated in a previous study on lung cancer patients in South Korea to minimize misclassification [26]. Third, long-term data were not available, as reimbursement for Osimertinib was effective from December 2017. However, because this study compared model structures under the same conditions, it was unlikely to have a significant impact on the results. Finally, T790M mutation status is expected to be different between the Osimertinib and PPC cohorts. In South Korea, Osimertinib is reimbursed only for patients with T790M-positive EGFR mutation, but PPC can be reimbursed regardless of the T790M mutation. However, prognostic role of the T790M mutation in EGFR-TKI rechallenge is unclear [27, 28], although several studies have reported that the T790M mutation is associated with poor prognosis in patients with NSCLC [29, 30].

## Conclusion

In this study, we developed a PSM and two STMs, with and without additional health states, to explore structural uncertainty through the comparison of different modeling approaches. Our findings demonstrate the considerable impact of incorporating additional health states such as brain metastases into economic models on estimates of incremental QALYs. These results underscore the importance of conducting comprehensive comparisons and justifications of modeling approaches in the economic evaluation of anticancer drugs. Our study suggests that additional health states with a heterogeneous QoL affect the outcomes of cost-effectiveness analysis in oncology.

### Electronic supplementary material

Below is the link to the electronic supplementary material.

Supplementary Material 1

## Data Availability

The datasets generated and/or analysed during the current study are not publicly available because the Korean Health Insurance Review and Assessment Service (HIRA) does not allow researchers to provide data personally or share publicly but are available from the corresponding author on reasonable request.

## Abbreviations

3-STM: 3-health state transition model
5-STM: 5-health state transition model
AIC: Akaike’s information criterion
AICc: Akaike’s information criterion with correction
BIC: Bayesian information criterion
BMIT: Brain metastasis with continuing initial therapy
BMST: Brain metastasis with subsequent therapy
EGFR: Epidermal growth factor receptor
EQ-5D: Euroqol 5-dimension
HIRA: Health Insurance Review and Assessment Service
HTA: Health technology assessment
ICD-10: International Classification of Disease-10th revision
NICE: National Institute for Clinical and Care Excellence
NSCLC: Non-small cell lung cancer
OS: Overall survival
PF: Progression-free
PFS: Progression free survival
PP: Post-progression
PPC: Pemetrexed plus platinum chemotherapy
PSM: Partitioned survival model
QALY: Quality-adjusted life-years
QoL: Quality of life
STM: State transition model
TKI: Tyrosine kinase inhibitor
TTNT: Time to next treatment
