# Supplementary material for: Comparison of partitioned survival modeling with state transition modeling approaches with or without consideration of brain metastasis: a case study of Osimertinib versus pemetrexed-platinum
Source: BMC Cancer. 2024 Feb 9;24:189. doi: 10.1186/s12885-024-11971-x (PMC10858528; doi:10.1186/s12885-024-11971-x)
Supplement: Supplementary file 1 — Supplementary Material 1 [file 12885_2024_11971_MOESM1_ESM.pdf]

Supplementary material: contents

Supplementary Methods S1: Data source

Supplementary Methods S2: Patient selection and propensity score-matching

Supplementary Methods S3: Method of extracting the date of death

Supplementary Methods S4: Transition probabilities and survival function

Supplementary Fig. S1. Study design of retrospective cohort study

Supplementary Fig. S2. Patient flow chart

Supplementary Table S1. Survival functions used in 3-STM

Supplementary Table S2. Survival functions used in 5-STM

Supplementary Table S3. List of utilities for one-way sensitivity analysis

Supplementary Table S4. Baseline characteristics of entire and matched cohort

Supplementary Table S5. Values for area under the curve by each model type

Supplementary Table S6. Mean LY and QALY estimates by each model type

Supplementary Table S7. Results of one-way sensitivity analysis with the same parametric distribution applied to both cohorts

Supplementary Methods S1: Database

We used national claims data from the Health Insurance Review and Assessment Service (HIRA) database in South Korea to analyse clinical outcomes. The database covers approximately 98% of the total population of Korea with a fee-for-service model and collects data for reimbursement from claims submitted by medical providers. It contains comprehensive data about demographics (such as age and sex), and healthcare services such as prescriptions, medical procedures, and records of diagnoses for approximately 50 million beneficiaries. Diagnoses are coded based on the International Classification of Disease-10th revision (ICD-10) (1).

The data from the National Health Insurance Service-national sample cohort (NHIS-NSC) version 2.0 for South Korea was utilized to adapt the operational definition, which was used to extract the date of death from the HIRA database, to improve accuracy. The NHIS-NSC version 2.0 includes approximately one million representative samples extracted by stratifying age, gender, participant's eligibility status, region, and income level from the total eligible Korean population (2). This dataset contains information on demographics, the month of death, and healthcare resource utilization, from 2002 to 2015.

Supplementary Methods S2: Patient selection and propensity score-matching

To select eligible patients from the claims data, we included newly diagnosed patients who had at least one inpatient or two outpatient claims with lung cancer according to the International Classification of Disease-10th revision (ICD-10) code (C34) during the index period (1 January 2010 to 30 October 2020). Newly diagnosed patients were identified by excluding patients with pre-existing lung cancer or other cancers and patients who received anticancer treatments within one year prior to the first diagnosis of lung cancer during the index period. Among these, we identified patients who initiated the first-line palliative therapy of EGFR-TKI (afatinib, gefitinib, erlotinib) and the date of first prescription was defined as the cohort entry date. Finally, patients who switched therapy to osimertinib were included in the osimertinib cohort, and patients who switched therapy to PPC were included in the PPC cohort.

Propensity score matching was conducted with the greedy 1:1 matching algorithm to alleviate the imbalance between the two cohorts. Propensity score was estimated through multivariate logistic regression analysis. As potential confounders, we considered age, sex, type of EGFR-TKI used as first-line therapy, duration of the EGFR-TKI, Charlson comorbidity index (CCI), and history of brain metastasis. Age and sex were extracted on the index date and CCI was computed over the 1-year pre-index period to assess patients' baseline comorbidity status (3, 4). The diagnosis of brain metastasis was defined as having at least one inpatient or two outpatient claims with ICD-10 code C793 (secondary malignant neoplasm of the brain and cerebral meninges). The history of brain metastasis was determined using claims data between the cohort entry date and the index date.

Supplementary Methods S3: Method of extracting the date of death

The date of death was extracted from the claims data by adapting an operational definition validated in a previous study. Jang et al. (2022) reported that the true-positive rate was over 98% and the false-positive rate was less than 2% in lung cancer patients of South Korea when the operational definition of the date of death is as follows: 1) death indication as a result of treatment or 2) the International Classification of Disease-10th revision (ICD-10) codes I46.1, R96, R98, or R99. 3) If there was no inpatient or outpatient medical record for 6 months, the patient was regarded as dead at the date of the last medical record (5). This operational definition has the potential to underestimate survival, given that some patients without an inpatient or outpatient medical record for 6 months may be alive for some time after the last medical record. Therefore, we added the median interval between the date of last medical record and the date of death among EGFR-mutated NSCLC patients without an inpatient or outpatient medical record for 6 months. The median interval was calculated using the data from the National Health Insurance Service-national sample cohort (NHIS-NSC) version 2.0 for South Korea. Since the database provides the month of death, it was assumed that the patients died on the 15th of the month of death. Details about the database are described in the Supplementary Methods S1: Database.

Supplementary Methods S4: Transition probabilities and survival function

The transition probabilities used in 3-STM are as follows:

$$TP(t, t-u) = \Pr(T \leq t | T > t-u) = 1 - \Pr(T > t | T > t-u)$$

$$= 1 - \frac{\Pr(T > t)}{\Pr(T > t-u)} = 1 - \frac{S(t)}{S(t-u)}$$

$$TP_1(\mathrm{t}, \mathrm{t}-\mathrm{u}) = 1 - TP_2(t) - \frac{S_{PF}(t)}{S_{PF}(t-u)}$$

$$TP_2(t, t-u) = 1 - \frac{S_{OSPF}(t)}{S_{OSPF}(t-u)}$$

$$TP_3(t, t-u) = 1 - \frac{S_{OSPP}(t)}{S_{OSPP}(t-u)}$$

where, $\mathrm{TP}(\mathrm{t}-\mathrm{u})$ is the transition probability at time t for cycle length u and Pr is the probability. The survival functions used in 3-STM are described in Supplementary Table S1.

Supplementary Table S1. Survival functions used in 3-STM

Survival function	Description
$S_{PF}(t)$	Predicted survival at time t remaining progression-free
$S_{OSPF}(t)$	Predicted survival at time t from progression-free to death<br>Outcome of interest is death and other events (i.e. progression) are treated as censored events
$S_{OSPP}(t)$	Predicted survival at time t from post-progression to death<br>Outcome of interest is death

The transition probabilities used in 5-STM are as follows:

$$TP_1(\mathrm{t,t-u}) = 1 - TP_2(t) - TP_4(t) - \frac{S_{PF}(t)}{S_{PF}(t-u)}$$

$$TP_2(t,t-u) = 1 - \frac{S_{OSPF}(t)}{S_{OSPF}(t-u)}$$

$$TP_3(t,t-u) = 1 - \frac{S_{OSPP}(t)}{S_{OSPP}(t-u)}$$

$$TP_4(t,t-u) = 1 - \frac{S_{BMPF}(t)}{S_{BMPF}(t-u)}$$

$$TP_5(t,t-u) = 1 - \frac{S_{BMPP}(t)}{S_{BMPP}(t-u)}$$

$$TP_6(t,t-u) = 1 - TP_7(t) - \frac{S_{PFMI}(t)}{S_{PFMI}(t-u)}$$

$$TP_7(t,t-u) = 1 - \frac{S_{OSMI}(t)}{S_{OSMI}(t-u)}$$

$$TP_8(t,t-u) = 1 - \frac{S_{OSMS}(t)}{S_{OSMS}(t-u)}$$

where, $\mathrm{TP(t-u)}$ is the transition probability at time t for cycle length u and Pr is the probability. The survival functions used in 5-STM are described in Supplementary Table S2.

Supplementary Table S2. Survival functions used in 5-STM

Survival function	Description
S_{PF}(t)	Predicted survival at time t remaining progression-free
S_{OSPF}(t)	Predicted survival at time t from progression-free to death<br>Outcome of interest is death and other events (i.e. progression) are treated as censored events
S_{OSPP}(t)	Predicted survival at time t from post-progression to death<br>Outcome of interest is death
S_{BMPF}(t)	Predicted survival in the PF health state at time t

	Outcome of interest is brain metastasis and other events (i.e. progression, death) are treated as censored events
$S_{BMPP}(t)$	Predicted survival in the PP health state at time t<br><br>Outcome of interest is brain metastasis and other events (i.e. death) are treated as censored events
$S_{PFMI}(t)$	Predicted survival in the BMIT health state at time t<br><br>Outcome of interest is disease progression or death
$S_{OSMI}(t)$	Predicted survival in the BMIT health state at time t<br><br>Outcome of interest is death and other events (i.e. progression) are treated as censored events
$S_{OSMS}(t)$	Predicted survival in the BMST health state at time t<br><br>Outcome of interest is death

PF, progression-free; PP, post-progression; BMIT, brain metastasis with continuing initial therapy; BMST, brain metastasis with subsequent therapy

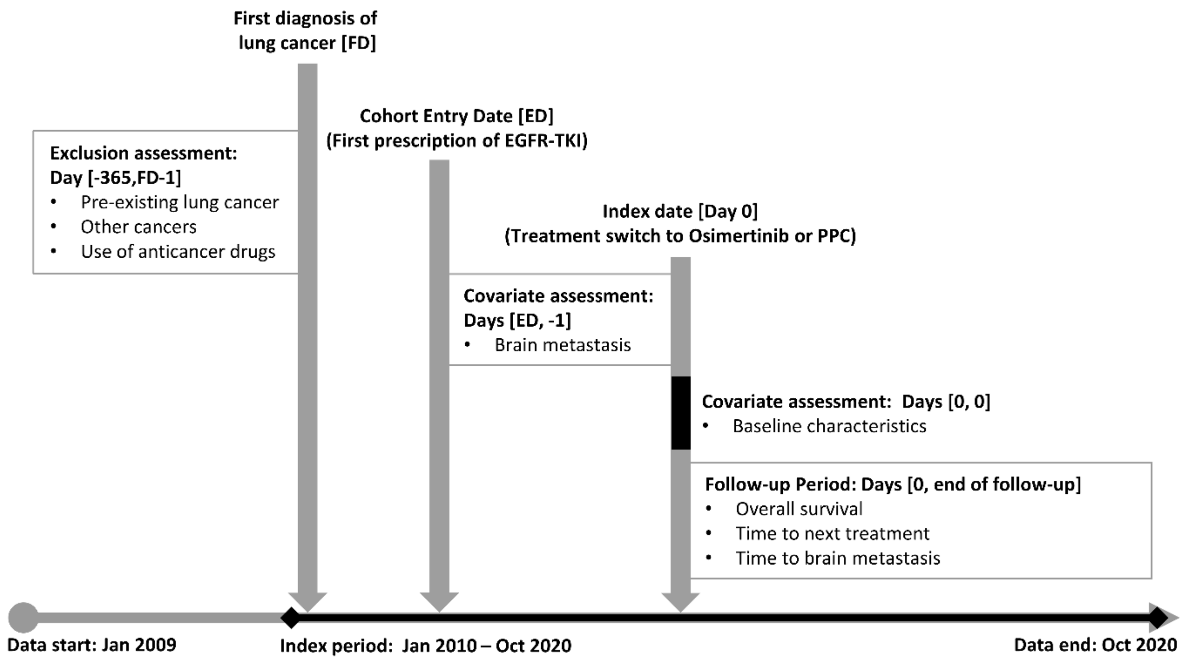

Supplementary Fig. S1. Study design of retrospective cohort study

EGFR, epidermal growth factor receptor; PPC, pemetrexed plus platinum chemotherapy; TKI, tyrosine kinase inhibitor

Supplementary Table S3. List of utilities for one-way sensitivity analysis

Model input	Osimertinib		PPC		Source
	Progression-free	Post-progression	Progression-free	Post-progression	
**Base-case**	0.800	0.758	0.730	0.688	Jiang et al.
**Sensitivity analysis for utilities**					
Bertranou et al.	0.805	0.715	0.778	0.715	Bertranou et al. (6)
AURA2 EQ-5D-5L Crosswalk Values	0.808	0.751	0.781	0.751	Bertranou et al. (6)
AURA2 EQ-5D-5L England Valuation Set Values	0.870	0.821	0.848	0.821	Bertranou et al. (6)

PPC, pemetrexed plus platinum chemotherapy

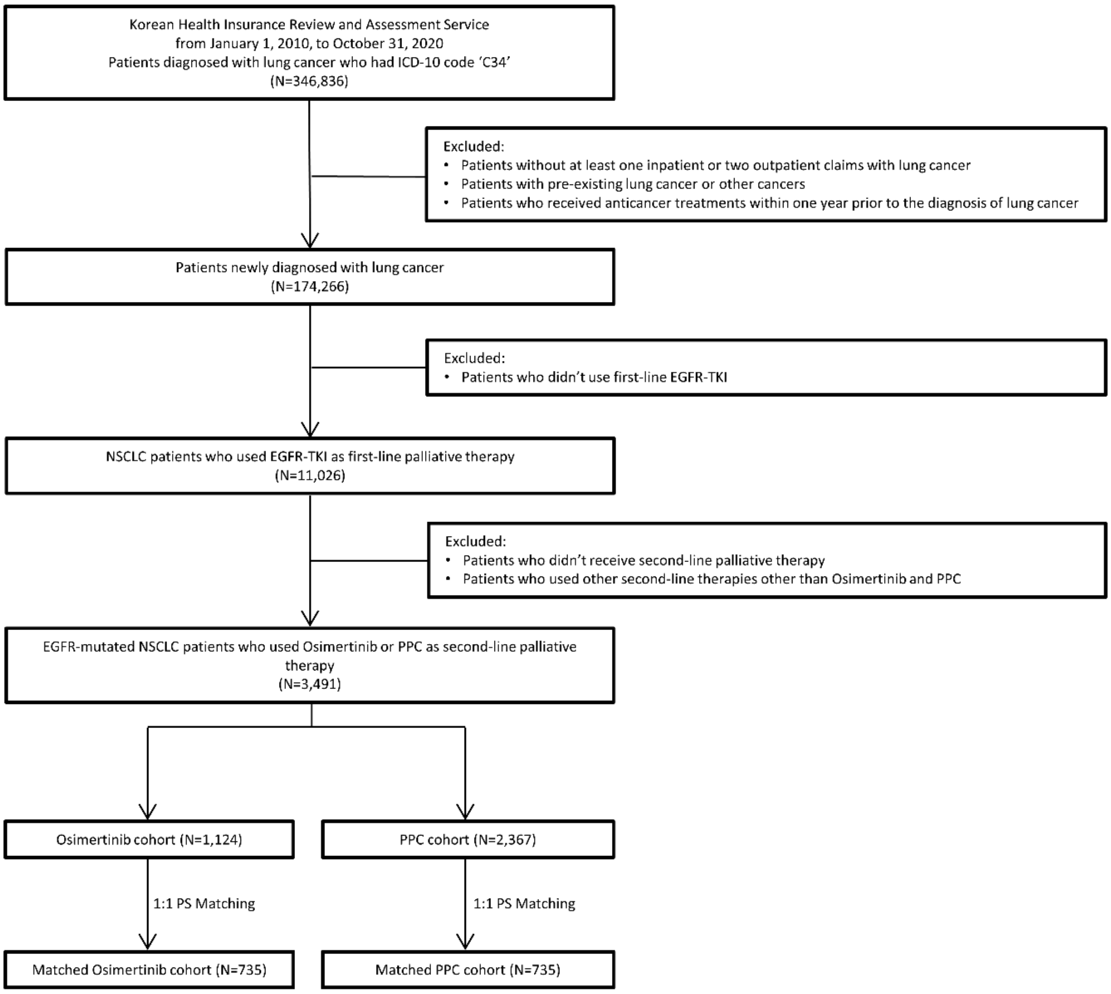

Supplementary Fig. S2. Patient flow chart

EGFR, epidermal growth factor receptor; ICD-10, International Classification of disease 10th revision; NSCLC, non-small cell lung cancer; PPC, pemetrexed plus platinum chemotherapy; PS, propensity score; TKI, tyrosine kinase inhibitor

Supplementary Table S4. Baseline characteristics of entire and matched cohort

	Entire cohort			Matched cohort		
	Osimertinib (n=1,124)	PPC (n=2,367)	SMD	Osimertinib (n=735)	PPC (n=735)	SMD
**Sex, n**			0.133			-0.011
Male	430	909		282	284	
Female	694	1,458		453	451	
**Age, mean**	65.06	64.46	0.053	64.51	65.23	-0.065
**Age group, n**			0.113			0.056
<60	357	787		248	238	
60–70	354	686		227	219	
70–80	291	693		190	208	
>80	122	201		70	70	
**CCI, mean**	5.62	6.04	-0.121	5.87	5.87	0.001
**CCI group, n**			0.144			0.019
0	101	131		51	53	
1–2	224	438		143	142	
3–4	90	203		54	57	
5	709	1,595		487	483	
**Brain metastasis**			0.005			-0.004
Yes	366	776		93	94	
No	758	1,591		642	641	
**First-line treatment, n**			0.279			0.017
Gefitinib	570	1,508		390	387	
Erlotinib	346	479		210	208	
Afatinib	208	380		135	140	
**Duration for first-line treatment, mean**	502.64	346.59	0.480	368.24	365.28	0.014

CCI, Charlson comorbidity index; SMD, standardized mean difference; PPC, pemetrexed plus platinum chemotherapy

Supplementary Table S5. Values for area under the curve by each model type

	Partitioned Survival Model		3-Health State Transition Model		5-Health State Transition Model	
	**Osimertinib**	**PPC**	**Osimertinib**	**PPC**	**Osimertinib**	**PPC**
Progression-free	1.969	0.735	1.969	0.735	1.841	0.762
Post-progression	0.577	0.816	0.669	0.901	0.806	0.870
Death	4.411	5.406	4.319	5.321	4.310	5.325

PPC, pemetrexed plus platinum chemotherapy

Supplementary Table S6. Mean LY and QALY estimates by each model type

	Partitioned Survival Model		3-Health State Transition Model		5-Health State Transition Model	
	**Osimertinib**	**PPC**	**Osimertinib**	**PPC**	**Osimertinib**	**PPC**
**Mean Life Years**	**2.400**	**1.511**	**2.486**	**1.587**	**2.499**	**1.590**
Progression-free	1.886	0.749	1.886	0.749	1.294	0.605
Brain metastasis with continuing initial therapy	-	-	-	-	0.483	0.171
Post-progression	0.515	0.762	0.600	0.838	0.337	0.529
Brain metastasis with subsequent therapy	-	-	-	-	0.385	0.285
**Mean QALYs**	**1.899**	**1.071**	**1.964**	**1.123**	**1.726**	**1.031**
Progression-free	1.509	0.547	1.509	0.547	1.035	0.442
Brain metastasis with continuing initial therapy	-	-	-	-	0.251	0.089
Post-progression	0.390	0.524	0.455	0.576	0.255	0.364
Brain metastasis with subsequent therapy	-	-	-	-	0.184	0.136

PPC, pemetrexed plus platinum chemotherapy; LY, life-year; QALY, quality-adjusted life-year
*Details may not add up to total due to rounding.

Supplementary Table S7. Results of one-way sensitivity analysis with the same parametric distribution applied to both cohorts

	Incremental QALY		
	**PSM**	**3-STM**	**5-STM**
**Base-case**	0.827	0.840	0.695
**Distribution of TTNT (PSM)**			
Log-logistic (best fit)	0.827		
Lognormal (Second best fit)	0.832		
**Distribution of OS* (PSM)**			
Generalized gamma (best fit)	0.827		
Log-logistic (Second best fit)	0.219		
**Distribution of TTNT (3-STM)**			
Log-logistic (best fit)		0.831	
Lognormal (Second best fit)		0.889	
**Distribution of OS* (3-STM)**			
Generalized gamma (best fit)		0.840	
Log-logistic (Second best fit)		0.219	
**Distribution of time to death from PF* (3-STM)**			
Log-normal (best fit)		0.840	
Log-logistic (Second best fit)		0.679	
**Distribution of time to death from PP (3-STM)**			
Log-normal (best fit)		0.845	
Log-logistic (Second best fit)		0.830	
**Distribution of TTNT from PF* (5-STM)**			
Generalized gamma (best fit)			0.695
Log-normal (Second best fit)			0.690

**Distribution of time to death from PF* (5-STM)**	
Log-normal (best fit)	0.695
Generalized gamma (Second best fit)	0.695
**Distribution of time to death from PP (5-STM)**	
Log-normal (best fit)	0.690
Exponential (Second best fit)	0.706
**Distribution of time to brain metastasis from PF (5-STM)**	
Generalized gamma (best fit)	0.648
Lognormal (Second best fit)	0.690
**Distribution of time to brain metastasis from PP (5-STM)**	
Exponential (best fit)	0.705
Log-logistic (Second best fit)	0.705
**Distribution of TTNT in metastasis with continuing initial therapy (5-STM)**	
Exponential (best fit)	0.700
Weibull (Second best fit)	0.688
**Distribution of time to death in metastasis with continuing initial therapy* (5-STM)**	
Log-normal (best fit)	0.695
Log-logistic (Second best fit)	0.678
**Distribution of time to death in metastasis with subsequent therapy (5-STM)**	
Log-normal (best fit)	0.683
Log-logistic (Second best fit)	0.676

* Dependent models were fitted as the assumption of proportional hazards held

3-STM, 3-health state transition model; 5-STM, 5-health state transition model; LY, life-year; OS, overall survival; PF, progression-free; PP, post-progression; PSM, partitioned survival model; QALY, quality-adjusted life-year; TTNT, time to next treatment

[Reference]

1. Kim JA, Yoon S, Kim LY, Kim DS. Towards Actualizing the Value Potential of Korea Health Insurance Review and Assessment (HIRA) Data as a Resource for Health Research: Strengths, Limitations, Applications, and Strategies for Optimal Use of HIRA Data. J Korean Med Sci. 2017;32(5):718-28.
2. Lee J, Lee JS, Park SH, Shin SA, Kim K. Cohort Profile: The National Health Insurance Service-National Sample Cohort (NHIS-NSC), South Korea. Int J Epidemiol. 2017;46(2):e15.
3. Quan H, Li B, Couris CM, Fushimi K, Graham P, Hider P, et al. Updating and validating the Charlson comorbidity index and score for risk adjustment in hospital discharge abstracts using data from 6 countries. Am J Epidemiol. 2011;173(6):676-82.
4. Quan H, Sundararajan V, Halfon P, Fong A, Burnand B, Luthi JC, et al. Coding algorithms for defining comorbidities in ICD-9-CM and ICD-10 administrative data. Med Care. 2005;43(11):1130-9.
5. Jang SC, Kwon SH, Min S, Jo AR, Lee EK, Nam JH. Optimal Indicator of Death for Using Real-World Cancer Patients' Data From the Healthcare System. Front Pharmacol. 2022;13:906211.
6. Bertranou E, Bodnar C, Dansk V, Greystoke A, Large S, Dyer M. Cost-effectiveness of osimertinib in the UK for advanced EGFR-T790M non-small cell lung cancer. J Med Econ. 2018;21(2):113-21.
